# Use of Wishart Prior and Simple Extensions for Sparse Precision Matrix Estimation

**DOI:** 10.1371/journal.pone.0148171

**Published:** 2016-02-01

**Authors:** Markku Kuismin, Mikko J. Sillanpää

**Affiliations:** 1 Department of Mathematical Sciences, University of Oulu, Oulu, Finland; 2 Biocenter, Oulu, Finland; National Institute of Environmental and Health Sciences, UNITED STATES

## Abstract

A conjugate Wishart prior is used to present a simple and rapid procedure for computing the analytic posterior (mode and uncertainty) of the precision matrix elements of a Gaussian distribution. An interpretation of covariance estimates in terms of eigenvalues is presented, along with a simple decision-rule step to improve the performance of the estimation of sparse precision matrices and associated graphs. In this, elements of the estimated precision matrix that are zero or near zero can be detected and shrunk to zero. Simulated data sets are used to compare posterior estimation with decision-rule with two other Wishart-based approaches and with graphical lasso. Furthermore, an empirical Bayes procedure is used to select prior hyperparameters in high dimensional cases with extension to sparsity.

## Introduction

Finding an alternative estimator for the covariance matrix is still an open problem in the field of statistics. For an extensive report on different reparametrizations of covariance matrices see [1] and [2]. Occasionally the inverse of the covariance matrix, the precision matrix, can be more useful in applications. Of particular note are the shrinkage-based methods which are able to coerce some elements of a precision matrix towards zero (e.g. [3], [4]). Estimation of a sparse precision matrix can be conceptualized as a way of approximating a Gaussian graphical model (GGM) for the data. In the GGM interpretation each variable represents a node in the network, and each non-zero off-diagonal element of a precision matrix creates an edge between the corresponding pair of nodes (variables).

The customary way to estimate precision matrix (represented by Θ = Σ^−1^, where Σ is the covariance matrix), is to maximize the log-likelihood of the data. With *n* normal distributed *p*-length vectors *Y*, this leads to a maximum likelihood estimate (MLE) $\hat{\Theta }={\left[\frac{1}{n}\overset{n}{\underset{i=1}{\sum }}\left(Y_{i}-\bar{Y}\right){\left(Y_{i}-\bar{Y}\right)}^{T}\right]}^{-1}$, where $\bar{Y}$ is the sample mean.

The maximum likelihood (ML) estimate can be considered a reliable estimate of a covariance matrix only when the fraction *p*/*n* is very small (e.g. [5]). Also, the ML-estimate does not generally have elements equivalent to zero. When the number of variables *p* exceeds the number of observations *n*, the ML-estimate will be singular and the maximum likelihood estimate of Θ cannot be computed.

There are at least two schools of thought pertaining to precision matrix estimation. One is to improve the precision or covariance matrix estimate, while ignoring the possible sparseness of the real Θ [6], [7], [5]. Another is to examine the sparse precision matrix estimate which can be used to examine the structure of the GGM [8], [3], [4], [9], [10]. This paper can be understood as an amalgamation of these schools: a method to improve the common Wishart prior is introduced, and a special decision-rule step is employed to gain a sparse posterior estimate for the precision matrix.

The graphical lasso [3], [11] is a well-favoured way of estimating a sparse precision and covariance matrix, respectively: Instead of maximizing the log-likelihood log(|Θ|) − *tr*(*S*Θ), where |.| is the matrix determinant, *tr*(.) is the trace of the matrix and *S* is the sample covariance, one should maximize penalized log-likelihood log(|Θ|) − *tr*(*S*Θ) − *ρ*||Θ||_1_, where *ρ* is non-negative penalization parameter and ||.||_1_ is the *L*_1_-norm, so that ${\left||\Theta |\right|}_{1}=\sum_{i}^{n}\sum_{j}^{n}\left|\theta_{ij}\right|$. This is a lasso-type of penalty for Θ[12] and will lead to a sparse estimate for Θ, which is positive definite even if *p* ≥ *n*. Recently [11] introduced a fast algorithm to solve the graphical lasso problem.

In the Bayesian framework, one can use a sparsity-inducing prior that shrinks elements of posterior estimate towards zero (see for example [4, 6]). The Wishart family of distributions is commonly used in multivariate analysis of Gaussian data to provide a convenient conjugate prior distribution for the precision and covariance matrices. Since the Wishart distribution is a conjugate prior for Θ it is analytically convenient, both because it provides positive definite posterior and is tractable. In this article the performance of the Wishart prior in the sparse context is also experimented with.

The structure of this paper is as follows: In section 2, it is shown that the posterior estimates of Θ and Σ can be represented as decompositions of sample covariance matrix eigenvalues and eigenvectors when the posterior of Θ is Wishart-distributed. A simple method based on the ideas of [5] is used to define a more accurate posterior estimate for the precision matrix. In section 3, the performance of the proposed Wishart prior method is examined using simulation studies similar to [4] and the graphical lasso is used as a baseline for comparison. The performance is measured with diagnostic loss measures. Convenient properties of Wishart distribution are illustrated and a decision-rule procedure is suggested to decide which of the off-diagonal elements of the precision matrix should be set to zero. In section 4, the methods are used in a network construction problem using data introduced by [13].

## Methods

Let *Y*_*i*_ ∼ *N*(*μ*, Θ^−1^) for *i* = 1, …, *n* be independent observations where Θ = (*θ*_*ij*_), called the precision matrix, is an inverse of a *p* × *p* covariance matrix Σ. Assume that *μ* is known and, without loss of generality, assume *μ* = 0. The likelihood function of the data $Y={\left(Y^{T},\ldots ,Y_{n}^{T}\right)}^{T}$ is

$$\begin{array}{c} p\left(Y|\Theta \right)=\prod_{i=1}^{n}p\left(Y_{i}|\Theta \right)\propto {\left|\Theta \right|}^{n/2}\exp\left\{-\frac{1}{2}tr\left(S\Theta \right)\right\}, \end{array}$$ (1)

in which Θ is a positive definite matrix (Θ ≻ 0) to be estimated. $S=\frac{1}{n}\sum_{i=1}^{n}Y_{i}Y_{i}^{T}$ is the maximum likelihood estimator (MLE) of Σ and, thus, *S*^−1^ is the maximum likelihood estimator of Θ. Here ^*T*^ denotes vector or matrix transpose. It is assumed that Θ could be a sparse matrix, having some off-diagonal elements exactly zero. With traditional maximum likelihood methods it is not possible to obtain sparse estimate. Additionally, the MLE is known to be reliable only when *p*/*n* ≪ 1 (see e.g. [14]). These problems can be alleviated by introducing prior information or by using a penalized likelihood approach [3], [4], [7], [9], [5].

### Wishart prior

Suppose *X* ∼ *N*(0, *B*) where *X* is *κ* × *p* matrix and *B* is a covariance matrix. Then *X*^*T*^ *X* ∼ *W*(*κ*, *B*) where *W*(*κ*, *B*) is the Wishart distribution with *κ* degrees of freedom and *B* is a positive definite symmetric scale matrix. The definition of *W*(*κ*, *B*) may be extended to allow arbitrary *κ* > *p* − 1 when it almost certainly has *rank* = *p*. The distribution is not defined for *κ* < *p* − 1. For *p* = 1, *W*(*κ*, 1) is identical with $\chi_{\kappa }^{2}$ distribution.

Wishart prior is set as a prior to the whole precision matrix. When Θ ∼ *W*(*κ*, *B*), the Wishart distribution has distribution function of the form

$$\begin{array}{c} p\left(\Theta \right)=\frac{1}{2^{\kappa p/2}}\frac{1}{{\left|B\right|}^{\kappa /2}\Gamma_{p}\left(\kappa /2\right)}{\left|\Theta \right|}^{\frac{\kappa -p-1}{2}}\exp\left\{-\frac{1}{2}\text{tr}\left(B^{-1}\Theta \right)\right\}, \end{array}$$ (2)

where *B* ≻ 0 is a *p* × *p* scale matrix, *κ* > *p* − 1 is a degrees of freedom parameter and Γ_*p*_(*κ*/2) is a multivariate gamma function. By the relationship between Wishart and inverse Wishart distributions, if a prior of Θ is chosen to be Wishart distributed with above parameters, then Σ = Θ^−1^ has inverse Wishart distribution Σ ∼ *W*^−1^(*κ*, *B*^−1^). Thus, the covariance matrix has a mean *B*^−1^/(*κ* − *p* − 1) where *κ* > *p* + 1 and variance is determined as $var\left(x_{ij}\right)=\kappa \left(b_{ij}^{2}+b_{ii}b_{jj}\right)$, in which *B* = (*b*_*ij*_) and *X*^*T*^ *X* = (*x*_*ij*_) (p. 24 in [15]).

Wishart distribution has several features which make it a viable choice. For example, the whole prior can be defined by just two parameters, thereby making it simple to use and meaning that it always leads to a positive definite posterior estimate for Θ. It is easily demonstrated that Wishart distribution has a mean equal to the product of degrees of freedom parameter *κ* and scale matrix *B*. Given that *κ* > *p* − 1 and *B*, the mode of the posterior distribution or the maximum a posteriori (MAP) is Θ_*MAP*_ = argmax_Θ_ *p*(Θ) = (*κ* − *p* − 1)*B*.

When the degrees of freedom parameter *κ* increases, the distribution of Θ concentrates around the scale matrix *B*. Use of smaller values for the degrees of freedom makes the distribution wider [16]. Later it will be shown that this is due to the shrinkage of the sample covariance matrix based on its eigenvalues.

The joint posterior density of precision matrix Θ is

$$\begin{array}{c} p(\Theta |Y)\propto p(\Theta )p(Y|\Theta ), \end{array}$$ (3)

where *Y* is a *n* × *p* data matrix drawn from *N*(0, Θ^−1^) distribution. When assigning the Wishart prior *W*(*κ*, *B*) to Θ, the posterior of Θ will also have Wishart distribution: Θ|*Y* ∼ *W*(*κ*+*n*, (*nS*+*B*^−1^)^−1^). The maximum a posteriori estimate, ${\hat{\Theta }}_{MAP}$ of this distribution has the analytic form

$$\begin{array}{c} {\text{argmax}}_{\Theta }\;p\left(\Theta |Y\right)=\left(\kappa +n-p-1\right){\left(nS+B^{-1}\right)}^{-1}, \end{array}$$ (4)

as well as posterior variance for the elements of $\hat{\Theta }$, $\text{var}\left({\hat{\theta }}_{ij}|Y\right)=\left(\kappa +n\right)\left(d_{ij}^{2}+d_{ii}d_{jj}\right)$, where (*nS*+*B*^−1^)^−1^ = (*d*_*ij*_), *i*, *j* = 1, …, *p*.

There has been some debate centered on whether to choose a weakly-informative or a non-informative prior. According to [17], non-informative prior should be considered as a starting point as it can easily be used to inspect the posterior in order to see if the latter makes sense. Weakly-informative prior should then be considered in cases of unexpected posterior.

In [17], the commonly used inverse Gamma distribution prior *inv-gamma*(*ϵ*, *ϵ*) is criticized for being sensitive to the choice of variance parameters, as *ϵ* → 0. Moreover [6] noted this tendency with inverse Wishart prior on a covariance matrix, as diagonal elements of Σ are inverse Gamma distributed.

In [18] and [16] it is argued that Wishart distribution may be too restrictive, as there are only *p*(*p* + 1)/2 + 1 distinct prior parameters and no parameters for modeling prior dependencies between the elements of Θ. For some subjective Bayesians this could mark Wishart as a far too inflexible choice. Nevertheless, as shown by [7] and [19], one can choose the hyperparameters of the Wishart distribution, with empirical Bayes procedures providing competing posterior estimates with no need for Markov Chain Monte Carlo (MCMC) sampling.

In principle, by following the work of [20] and [6], a hierarchical model could be built by setting our own prior for parameters *κ* and *B* in Eq 2. However, this would necessitate tuning the parameters on the next layer in the model hierarchy, thereby requiring one to then tune the hyperparameters of these priors. As mentioned by [4], the choice of hyperpriors is not trivial and depends upon the sample size; when *p*/*n* > 1, more informative hyperprior has to be used to impose shrinkage to the precision matrix elements. Additionally, MCMC sampling would need to be used for estimation, rather than more rapid analytic formulas.

### Eigenvalues of the estimated precision matrix

Before choosing which elements of the posterior estimate are zero, one should verify if there is a way to gain a more reliable “starting value” for the sparse estimate of Θ. It may be difficult to make the aforementioned decision if the estimate is biased before the sparsity is induced in the final sparse estimate.

In practice, it is impossible to fully monitor whether every diagonal and off-diagonal element of the posterior estimate of Θ is realistic. However, it is an easier task to examine the *p* eigenvalues of the estimate, denoted by *λ*_1_ ≥ *λ*_2_ ≥, …, ≥ *λ*_*p*_. When the ratio *p*/*n* is greater than one, it is known that *S* is singular and has zero as its eigenvalue. Also, small eigenvalues of *S* are near zero and can be slightly negative. Clearly, every *λ*_*k*_, *k* = 1, …*p* has to be larger than zero to obtain a positive definite posterior estimate for Θ.

**Fig 1:**
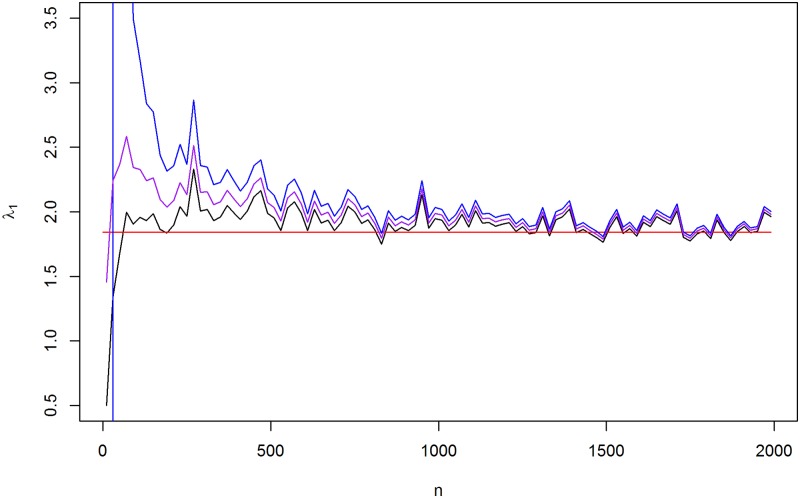
The shrinkage of the largest eigenvalues. The largest eigenvalue of MAP (black line), ML-estimate (blue line), posterior mean (purple line) and the real eigenvalue of Θ (red line) when the data is sampled from N(0, Θ^−1^) distribution with sample size *n* and *p* = 20.

Here the properties of the Wishart prior of form *W*(*r*, *α*^−1^ *I*), which leads to a ridge-type posterior estimate, are demonstrated. It is easily shown that the posterior estimate of Θ can be written as a linear shrinkage estimator, based on the eigenvalues of the maximum likelihood estimate. The MLE or the sample covariance has eigenvalue decomposition as *S* = *XLX*^*T*^, where *L* = *diag*(*l*_1_, …, *l*_*p*_) is a diagonal matrix with eigenvalues on the diagonal and *X* is the *p* × *p* orthogonal matrix whose *i*^*th*^ column is the eigenvector that corresponds to the eigenvalue *l*_*i*_. The MAP-estimate or the posterior mean is *a*(*nS* + *αI*)^−1^, where *a* is a constant *n* + *r* − *p* − 1 for the MAP-estimate or *n* + *r* for the posterior mean. It can be written as *δ*(Φ) = *X*Λ*X*^*T*^ = *XΦ*(*l*)*X*^*T*^, Φ(*l*) = *diag*(Φ(*l*_1_), …, Φ(*l*_*p*_)), $\Phi \left(l\right)=\frac{a}{nl+\alpha }$, which is a special case of ridge-type shrinkage of the eigenvalues. As can be seen from Φ(*l*), when the small hyperparameter value *r* is used to choose uninformative prior for Θ, less shrinkage is provided for eigenvalues of the sample covariance matrix. The shrinkage function Φ(*l*) approaches the MLE-based eigenvalue 1/*l* when *p* is fixed and *n* increases. This systematic shrinkage of the eigenvalue is illustrated in Fig 1, where the red line indicates the largest eigenvalue of the real Θ. The ML-estimate tends to amplify the largest eigenvalue even when *n* ≫ *p*. For eigenvalues of a covariance matrix with a different choice of the scale matrix, see [20].

**Fig 2:**
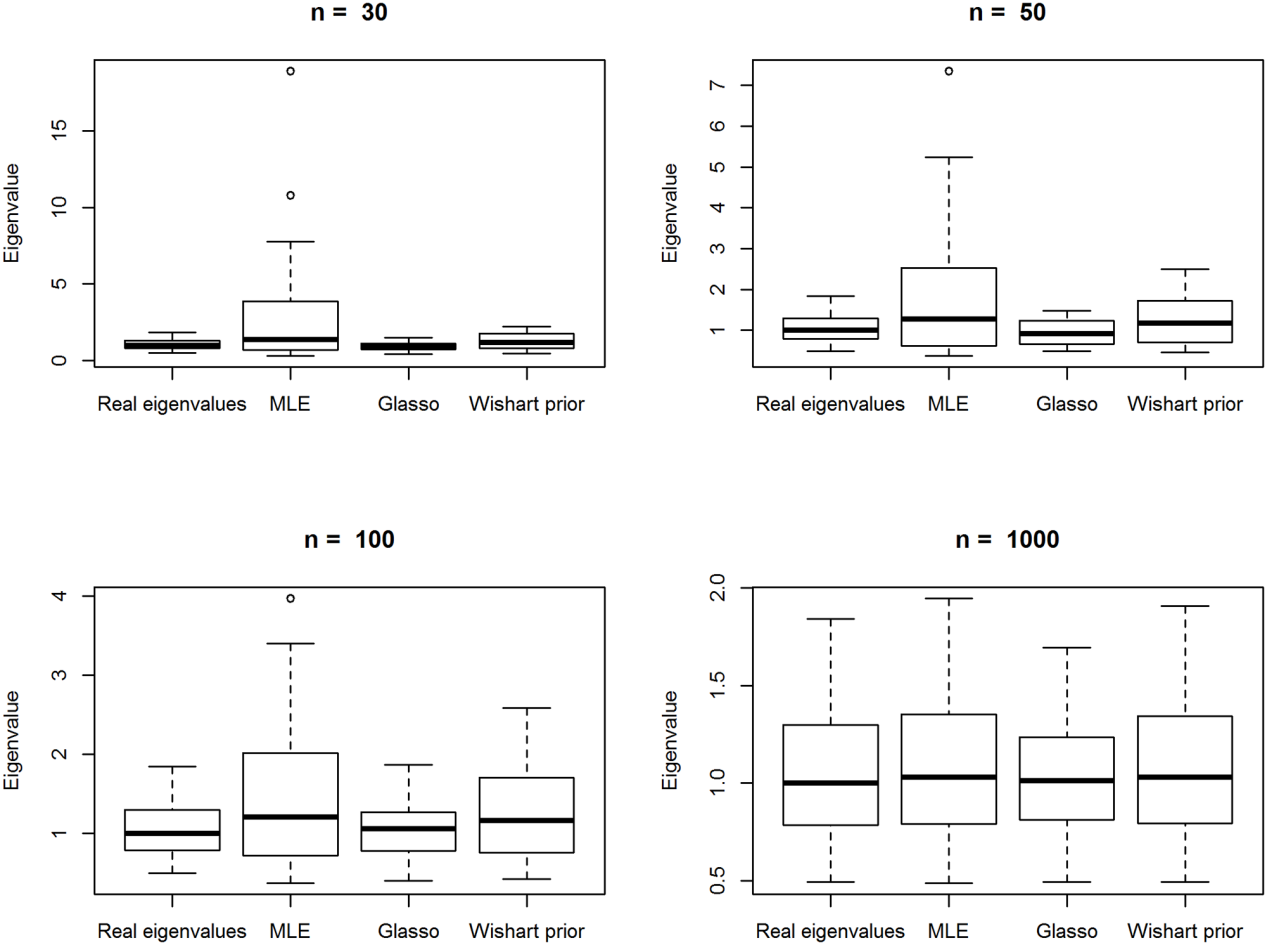
Boxplots of the eigenvalues. From left to right: The eigenvalues of the real precision matrix, the maximum likelihood estimate (MLE), graphical lasso (Glasso, *ρ* chosen with 5-fold cross-validation) and MAP of Wishart *W*(2*p*, 1/2*pI*) prior when the true precision matrix is sparse (as described in section 3) with sample sizes of 30, 50, 100 and 1000.

As can be seen from Fig 2, the MLE seems to stumble with the larger eigenvalues. This is also noted by [20]. Graphical lasso and MAP-estimate are both able to shrink the largest eigenvalues of the ML-estimate and thus correct the instability of the eigenvalues. As the sample size grows, eigenvalues of the estimators move closer to the real eigenvalues. The graphical lasso with cross-validation seems to shrink the eigenvalues quite efficiently and even seems to “over-shrink” the largest eigenvalue. It is unclear whether, the MLE, MAP or the graphical lasso estimate of the precision matrix converges fastest towards the real eigenvalues.

**Fig 3:**
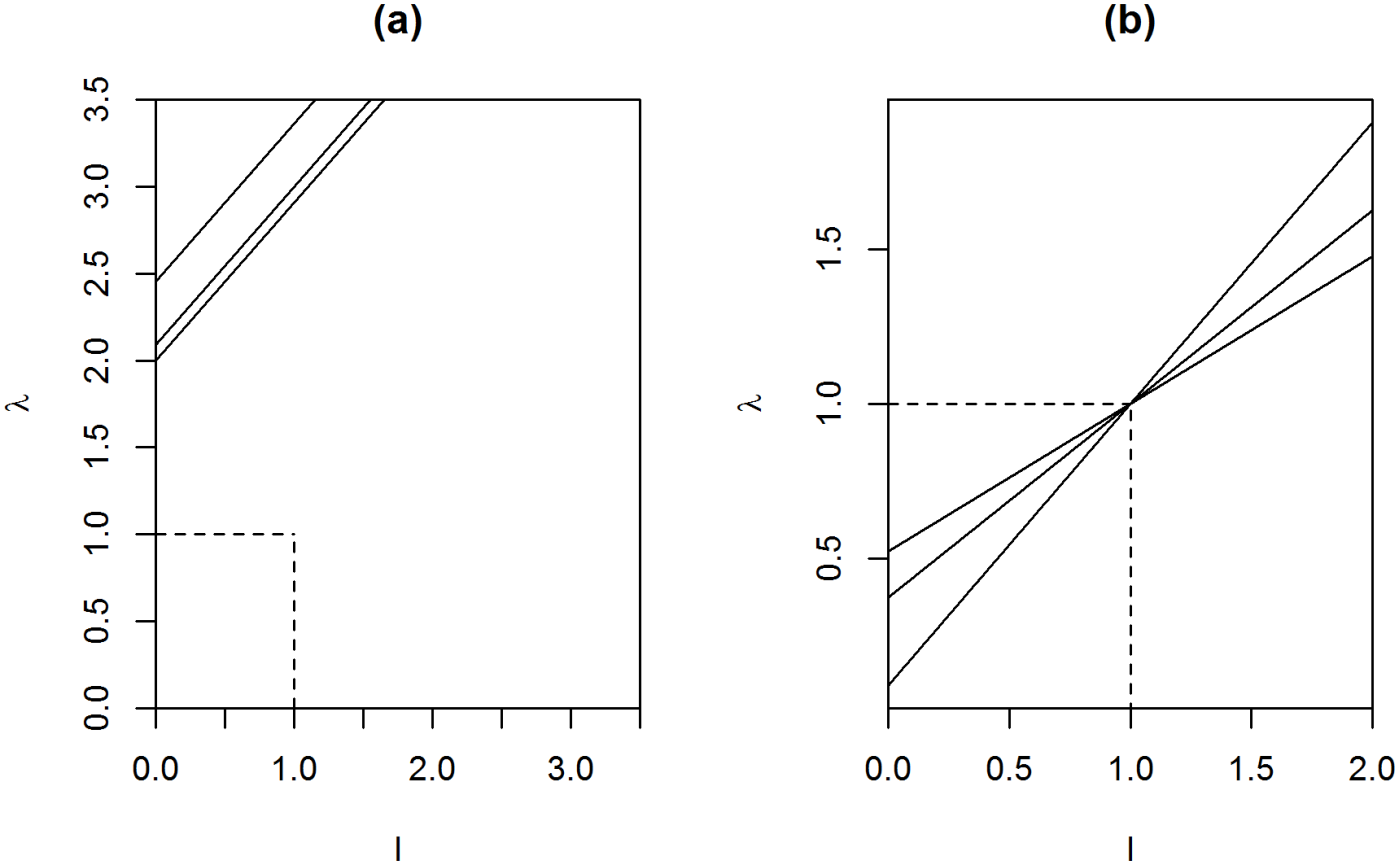
Linear shrinkage of the eigenvalues. Shrinkage of the eigenvalues of the MAP of the covariance matrix, as *r* is fixed and *α* changes when *n* < *p* (a) and with *α* = *r* − *p* − 1 as the sample size *n* increases (b). The x-axis denotes the eigenvalues of the MLE (*l*) and y-axis denotes the corresponding eigenvalues of the posterior estimate (*λ* = Φ(*l*)^−1^).

**Fig 4:**
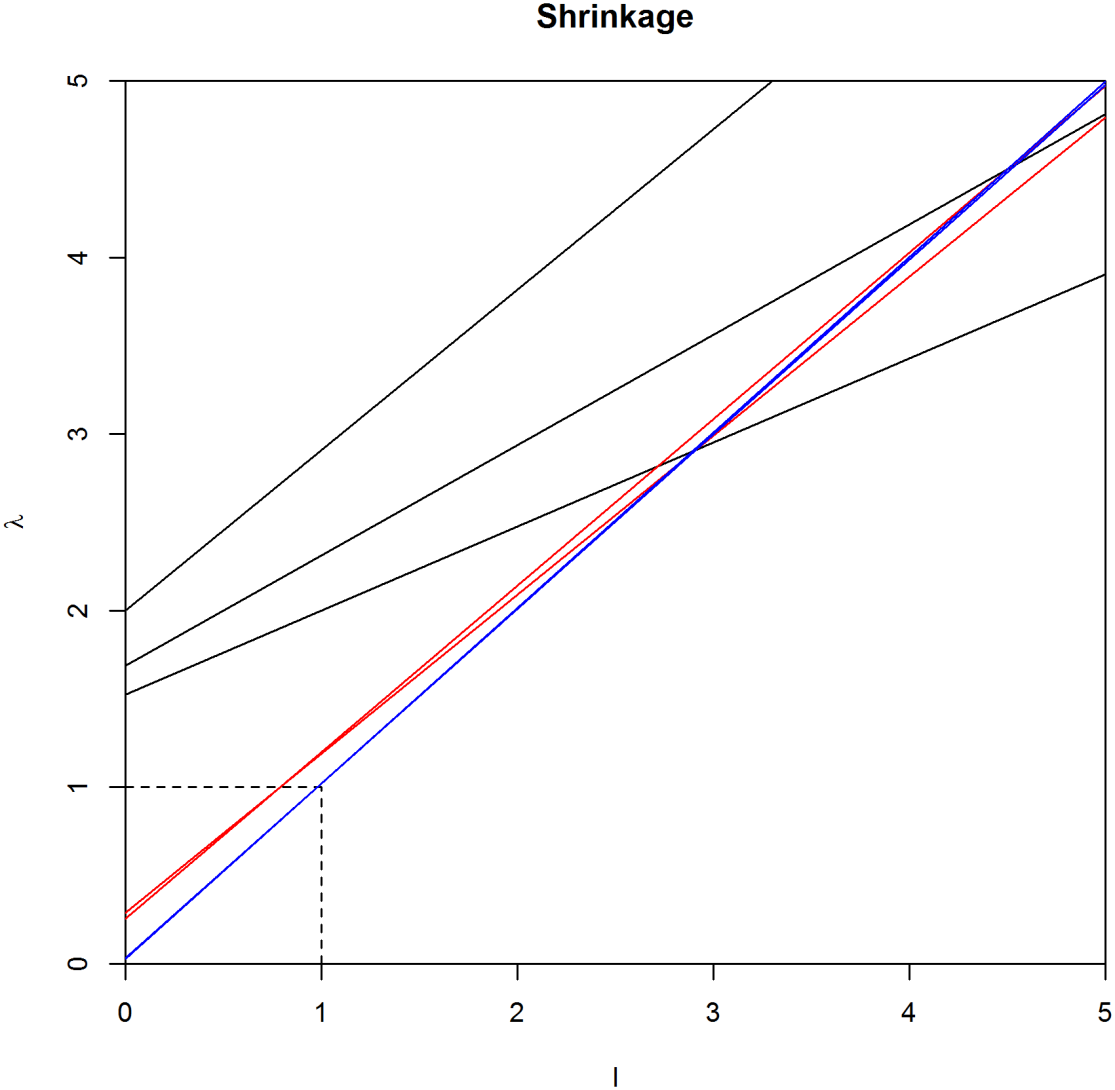
Linear shrinkage of the eigenvalues. Shrinkage of the eigenvalues of the MAP of the covariance matrix as *r* = *α*. When *n* < *p*, all eigenvalues increase when *r* is small but when *r* increases, the increment gets more moderate (black lines). When *n* increases, shrunken eigenvalues get closer to the sample eigenvalues and the growth of *r* does not significantly affect the estimated eigenvalues (red lines). When *n* ≫ *p*, estimated eigenvalues start to coincide to the sample eigenvalues (blue lines).

Using the fact that the estimator for the covariance matrix is just the decomposition of the Φ(*l*) inverse with the same eigenvectors as the MLE, the linear type shrinkage of the eigenvalues of the covariance matrix is $\frac{n}{a}l_{i}+\frac{\alpha }{a}$, *i* = 1, …, *p*. Instead of the eigenvalues of the precision matrix, we take a look at the covariance matrix eigenvalues. This is because it is much easier to examine the linear shrinkage of the sample covariance matrix eigenvalues *l*_*i*_, which are always greater than or equal to zero, than the eigenvalues of the ML-based precision matrix estimate, which are not always determined. When *n* < *p*, the difficulty of selecting a proper scale parameter *α* arises from the problem of choosing a value that shrinks large eigenvalues and increases the smaller ones to make the estimate positive definite. In Fig 3a it can be seen that when *α* increases, *α* ≥ *r*, all sample eigenvalues grow when *n* < *p* and the increment becomes greater, in accordance with *α*. In cases when *S* is close to singularity this may lead to better results as small sample eigenvalues increase dramatically. Another possibility is to choose scale parameter *α* as *r* − *p* − 1 for the MAP of Σ and thus for Θ. With this choice of *α*, the Wishart prior increases sample eigenvalues lesser than one and shrinks eigenvalues greater than one. Also, the stable eigenvalues (*l*_*i*_ = 1) do not change. In this case, when the sample size increases the shrunken eigenvalues approach the sample eigenvalues, which is desirable when *n* ≫ *p*. This is presented in Fig 3b, where the line denoting the shrinkage function Φ(*l*)^−1^ = *λ* becomes steeper when *n* increases. We may posit that this feature makes it a safe choice when *n* ≫ *p*. However, the best choice could be a compromise between these two options for the relation of *r* and *α*. Thus, one can choose hyperparameters *r* and *α* as equal. This case is illustrated in Fig 4.

### Choosing the degrees of freedom parameter

The choice of the degrees of freedom parameter *r* for the Wishart prior *W*(*r*, *α*^−1^ *I*) determines how much shrinkage is induced to the eigenvalues of a sample covariance matrix *S* when calculating the MAP. In [5], it is proposed that the condition number of the covariance matrix estimate should be restricted by some values in order to gain a stable, well-conditioned estimate. The condition number of the MAP-estimate is defined as *cond*(Θ_*MAP*_) = *λ*_1_/*λ*_*p*_, where *λ*_1_ is the largest and *λ*_*p*_ the smallest eigenvalue of Θ_*MAP*_. Clearly, the ratio *λ*_1_/*λ*_*p*_ is very large when the MAP-estimate is close to singularity and, by increasing the small eigenvalues, may lead to better estimate. The value of *α* is set as *α* = *r*. The condition number can be monitored and *r* increased until the *cond*(Θ_*MAP*_) is smaller than a predetermined value. More informative prior should be used when *n* ≤ *p* because the estimate is assumed to be sparse and the sample covariance matrix unreliable. In our numerical experiments, the restricted condition number was chosen to be smaller than $\text{cond}\left(\Theta_{MAP}\right)-2/\sqrt{n}$, where the value of $2/\sqrt{n}$ diminishes as the number of observations increases, leading back to use of non-informative Wishart prior. It should be noted that in high dimensional case, small eigenvalues of the sample covariance are very close to zero and posterior eigenvalues are approximately *α*/(*n* + *r* − *p* − 1). Thus, it may be concluded that they are determined by the sample size and dimension, when *α* and *r* are set as uninformative.

### Decision-rule for sparse precision matrix estimation

With a ridge-type posterior estimate of the form *a*(*nS* + *B*^−1^)^−1^, it is impossible to set elements of estimated precision matrices exactly to zero, with any kind of choice in the degrees of freedom parameter of the Wishart prior. This will always be a problem when using continuous prior distributions, because the resulting posterior probability of {*θ*_*ij*_} being zero is non-existent. Therefore, some external procedure should be used to set appropriate off-diagonal elements exactly to zero. We propose a decision-rule process based on thresholding the smallest elements in the precision matrix in a stepwise manner using extended Bayesian information criterion (EBIC) to produce a sparse posterior estimate of Θ.

Suppose we are interested in gaining a sparse posterior estimate for Θ. As mentioned by [21], the Bayesian information criterion (BIC) tends to produce dense graphs in high dimensions. We have also faced similar problems in other simulation studies and in empirical data studies outside of this work. Thus we propose to use the EBIC instead of the BIC for the decision-rule to choose the sparsity level of the estimated Θ. This usually leads to more sparse estimate for the precision matrix and thus sparser but still consistent graph topology for Gaussian graphical models. For more about EBIC, see [22].

The EBIC is of the form

$$EBIC=-n[\log(|\hat{\Theta }\left|\right)-\text{tr}\left(\hat{\Theta }S\right)]+\text{df}\left(\hat{\Theta }\right)\log n+4\text{df}\left(\hat{\Theta }\right)\gamma \log p,$$

where $\hat{\Theta }$ is the sparse posterior estimate of Θ and $\text{df}(\hat{\Theta })$ is the number of non-zero elements in $\hat{\Theta }$, $\log(|\hat{\Theta }\left|\right)-\text{tr}\left(\hat{\Theta }S\right)$ is the unnormalized log-likelihood and *γ* is a user specified parameter which can be set as 0.5 as a default [22]. Here, the smaller value of EBIC indicates its better fit for the model.

We use an universal thresholding method to induce sparsity to the Bayes estimate of Θ. We apply a stepwise selection and pick the posterior estimate with maximum level of sparseness, which minimizes the EBIC. The decision-rule works in the following manner.

Let *A* = (*a*_*ij*_) be a symmetric matrix where elements are the absolute values of the posterior estimate of the precision matrix Θ, $a_{ij}=\left|{\hat{\theta }}_{ij}\right|$ for all *i*, *j* = 1, …, *p*.

The non-zero elements of $\hat{\Theta }$ are obtained by using the following algorithm:

1. Set the *p*(*p* − 1)/2 off-diagonal elements of *A* in ascending order as a vector *a*^(1)^ ≤ *a*^(2)^ ≤ … ≤ *a*^(*p*(*p* − 1)/2)^.
2. Initialize *l* = 1.
3. If *a*_*ij*_ ≤ *a*^(*l*)^, set ${\hat{\theta }}_{ij}={\hat{\theta }}_{ji}=0$, *i* ≠ *j* and set ${\hat{\Theta }}_{l}=\left({\hat{\theta }}_{ij}\right)$ for all *i* = 1, …, *p* − 1, *j* = (*i*+1), …, *p*, *i* ≠ *j*.
4. Calculate the EBIC for the ${\hat{\Theta }}_{l}$.
5. Set *l* = *l*+1.
6. Return to step 3, or stop if stopping criterion, e.g. *l* = *p*(*p* − 1)/2 + 1, is met.

Select such ${\hat{\Theta }}_{k}$ as the sparse posterior estimate of Θ which minimizes EBIC. We did not apply any additional stopping criterion for the algorithm in our studies. For a large *p* this is a greedy procedure but it can be executed in less than *p*(*p* − 1)/2 steps by using e.g. pre-defined threshold values or examining the monotonicity of the EBIC by plotting it against the number of non-zero elements of the sparse posterior estimate corresponding to the value of EBIC.

We are more interested in of these kind of methods, which are easy to run with minimal adjustment of any model value whatsoever. This approach is straightforward and has some similarities with the special thresholding, which can be applied to the entries of the sample covariance matrix in the graphical lasso algorithm [23], [11]. These expansions can be wrapped around the graphical lasso to gain a boost in the execution time. This resembles our method but with pre-defined threshold values.

With a moderate sample size covering a couple of hundred of variables, this is a feasible design and is invariant under permutation of variables. This procedure can be used in any Bayes estimate of the precision matrix. Without any mathematical justification we will show in numerical examples that this procedure works surprisingly well in both risk minimization settings and in data analysis and does not violate the positive definiteness restriction of $\hat{\Theta }$.

It should be noted that setting some elements to zero may lead to a non-positive definite posterior estimate of Θ. In this case, a suitable value *δ* > 0 can be chosen to make $\hat{\Theta }$ positive definite by adding it to the diagonal of $\hat{\Theta }$. Also, because the same threshold is used for all the precision matrix elements in our stepwise decision-rule procedure, the variables should be standardized to the same scale.

## Simulation study

We were interested to see how the sample size affect the posterior estimate and the performance of our method when using the simple Wishart prior. To measure the performance of the analytic Bayesian procedure of using *W*(*r*, *α*^−1^ *I*)-prior together with the aforementioned decision-rule, 50 simulated data sets from *N*(0, Θ^−1^) distribution were generated. Three unstructured and three structured matrices for Θ and Σ were considered in following fashion.

1. A diagonal matrix with elements randomly sampled from Uniform distribution; *θ*_*kk*_ ∼ *Uniform*(1, 1.25).
2. An autoregressive AR(1) matrix for Σ = (*σ*)_*kk*′_ with *σ*_*kk*′_ = 0.7^|*k* − *k*′|^.
3. An AR(2) matrix with *θ*_*kk*_ = 1, *θ*_*kk* − 1_ = *θ*_*k* − 1*k*_ = 0.5 and *θ*_*kk* − 2_ = *θ*_*k* − 2*k*_ = 0.25.
4. A sparse matrix for Θ with at least 80% off-diagonal elements set to zero.
5. A moderate matrix for Θ with at least 40% off-diagonal elements set to zero.
6. A dense matrix for Θ with less than 5% off-diagonal elements set to zero.

A Cholesky decomposition was used to generate the three unstructured positive definite matrices described in d), e) and f), following the approach of [4]. The dimension *p* was fixed to 20 and *n* was chosen to be 10, 20, 50 and 100 for each of the 50 data sets.

### Performance measures

Three different loss functions were used to compare the risks of the estimated precision matrix $\hat{\Theta }$. The Kullback-Leibler loss (K-L) or the entropy loss, defined as $\text{KL}\left(\Theta ,\hat{\Theta }\right)=\text{tr}\left(\Theta^{-1}\hat{\Theta }\right)-\log(|\Theta^{-1}\hat{\Theta }\left|\right)-p$, the *L*_2_-loss (*L*_2_) defined as $L_{2}\left(\Theta ,\hat{\Theta }\right)=||\Theta -\hat{\Theta }{\left|\right|}_{F}$ and the quadratic loss (Ql) defined as $Q\left(\Theta ,\hat{\Theta }\right)=\text{tr}{\left(\Theta^{-1}\hat{\Theta }-I\right)}^{2}$. All measures approach zero as the estimate $\hat{\Theta }$ approaches the real precision matrix.

We used three other estimators for both Σ and Θ as baselines to compare the performance of our decision-rule with prior having diminished condition number for Θ.

- Ledoit and Wolf estimator [19]. This is a frequentist estimator, or an empirical Bayes method where data driven parameters are used in the inverse Wishart prior assigned to Σ. This produces a non-sparse shrinkage estimate for Σ. We invert this estimate to get the estimate of Θ.
- Bouriga and Féron model 1 [20]. This is a hierarchical Bayes model for Σ where flat improper priors are assigned to both degrees of freedom and the scale matrix of the inverse Wishart distribution. This will lead to a ridge-type non-sparse posterior estimate for Σ. The estimator of Σ with respect to the squared Frobenius loss function (see [20]) was used in this simulation study. We calculated 10000 iterations with the Metropolis-within-Gibbs algorithm among which 5000 of the first MCMC samples were ignored as the burn-in period. Again the posterior estimate of Σ is inverted to gain the estimate of Θ.
- Graphical lasso (Glasso) is currenty considered as a state-of-art approach for sparse precision and covariance matrix estimation within the frequentist framework. Thus, “glasso” R package [3] based on the algorithm 2 of [11] was used as a baseline to compare our results with each matrix and data combination. The regularization parameter *ρ* needed in the Glasso algorithm was chosen by EBIC as described earlier. The candidate sequence for *ρ* was chosen as [0.01, 0.02, 0.03, …, 0.5].

For both our method and Glasso the default value of *γ* = 0.5 was used in this simulation study for the EBIC. All methods were run with R but the analysis concerning the Bouriga and Féron posterior estimate was performed with MATLAB.

For our estimator, the MAP was used as the point estimate of the precision matrix, because it induces more shrinkage to the eigenvalues than the posterior mean. The mean and standard deviation were calculated for each loss function, based on 50 different data replicates to compare these frequentist risk measures. See [24] for loss measures in MCMC sampling.

## Results

**Fig 5:**
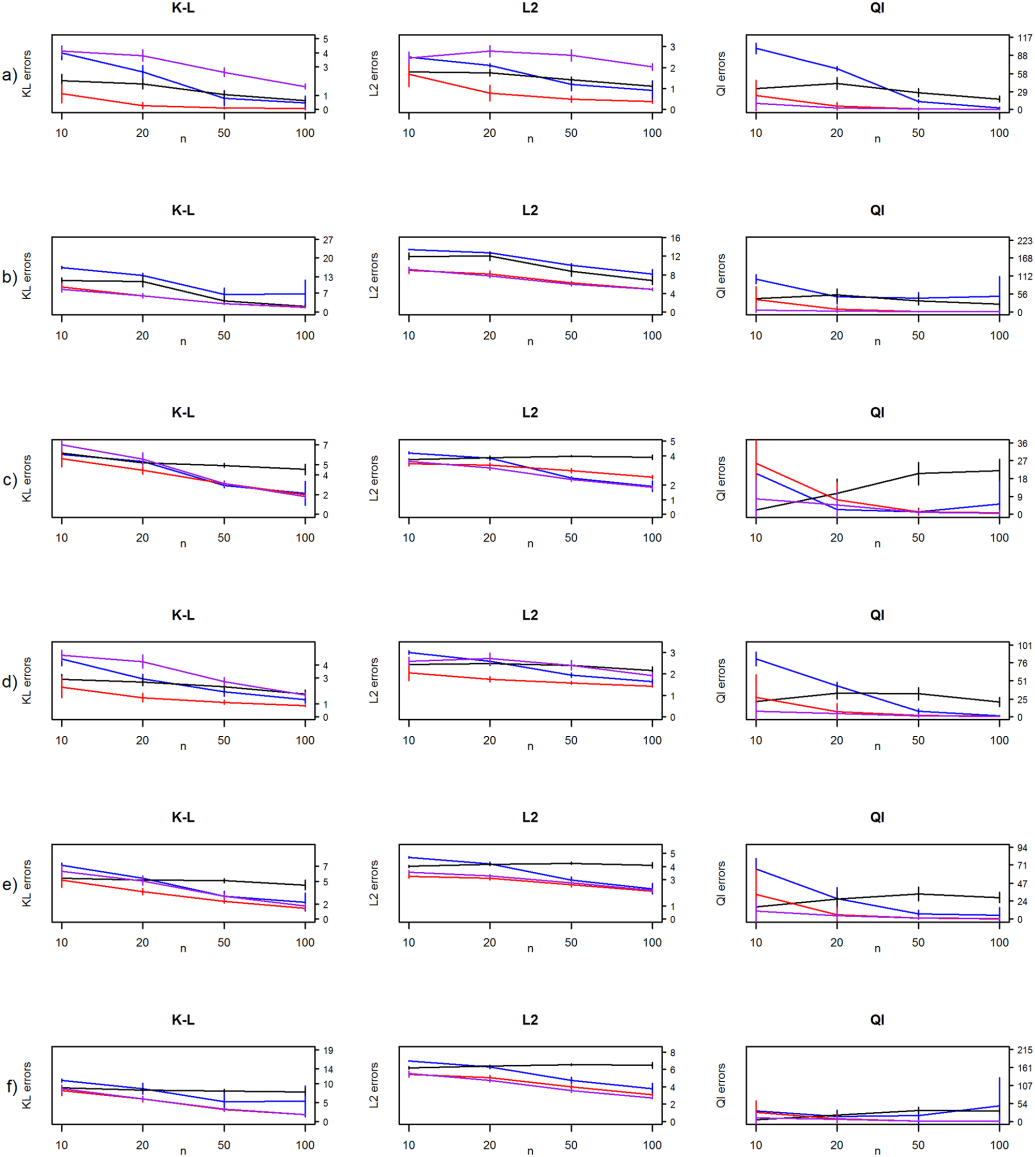
Frequentist risk estimates from the simulation studies. Kullback-Leibler (K-L), L2-loss (L2) and quadratic losses (Ql) for MAP-estimate with diminished condition number combined with decision-rule (blue line), hierarchical Bayes model by Bouriga and Féron (purple line), the Ledoit and Wolf estimate (red line) and graphical lasso algorithm (black line) for (a) diagonal, (b) AR(1), (c) AR(2), (d) sparse, (e) moderate and (f) dense precision matrices.

The risk measures are illustrated in Fig 5. With a small *p*/*n* ratio all methods seem to perform well. Unless the ratio *p*/*n* is minuscule the eigenstructure tends to be systematically distorted, resulting in a biased estimate for Θ. All methods give estimates with relatively high risk when *p* ≥ *n*. This is consistent with the Tuckey’s 5^k^ rule (see e.g., p. 35 in [25]) which suggest that at least 5^k^ observations are needed to estimate the k^th^ moment. Otherwise, in the cases c, d, e and f all other methods seem to outperform Glasso when *n* > *p*, although the difference is not always that clear.

We are surprised by the moderate performance of the graphical lasso in this simulation setting. Even when the sample size increases, the risk measures do not diminish, and that is quite unexpected. This is most certainly due the EBIC used to choose the regularization parameter *ρ*. Still this is out of tune with our method in which performance is more consistent with the sample size. Both the MAP-estimate with diminished condition number combined with our decision-rule and Glasso stumble with AR(1) and dense precision matrices. Furthermore, the true matrices of these models have the highest condition numbers, at 28.55 and 28.94 respectively.

The dark horse in this simulation study is the Ledoit and Wolf estimator which gives the smallest frequentist risk with all measures in almost all cases. It is also clearly the fastest method compared to the others. The Bouriga and Féron model provides competent estimates and particularly the lowest risk for the quadratic loss. The only problem with this approach is the time which is used in the Gibbs sampling even with our moderate MCMC sample size. This is the Achilles’ heel of the hierarchical Bayesian models in the covariance and precision matrix estimation where the time and computing power needed for the analysis is a common problem. For example [9] reported a computation speed of about a minute for a block Gibbs sampler for *p* = 100. Our method takes mere seconds to perform in the same dimension.

**Fig 6:**
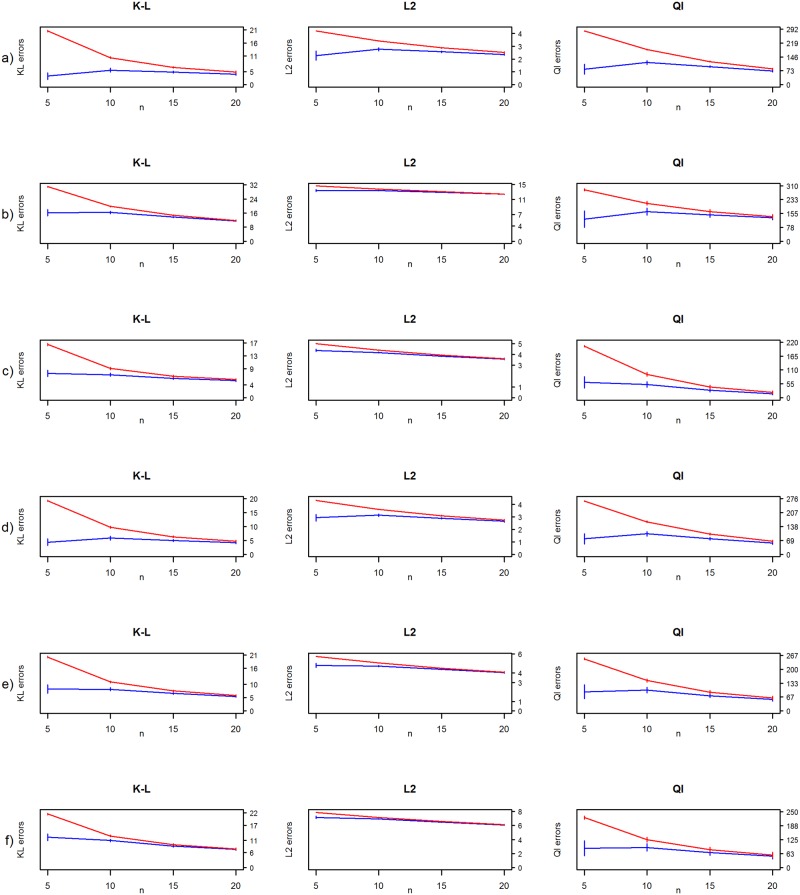
Frequentist risk estimates from the simulation studies. Kullback-Leibler (K-L), L2-loss (L2) and quadratic losses (Ql) for (a) diagonal, (b) AR(1), (c) AR(2), (d) sparse, (e) moderate and (f) dense precision matrices for diminished condition number (blue line) and common Wishart prior based MAP (red line) with *r* and *α* set as *p*.

In Fig 6, weakly-informative prior is compared to uninformative prior without decision-rule. The Wishart prior where the degrees of freedom parameter was set to be weakly-informative outperforms the uninformative Wishart prior in all cases when *n* ≤ *p*, and performs at a similar level when there are more data points. This is in line with the suggestions of [17]. Of particular note is the fact that the performance measures under quadratic loss decrease in every case. In our numerical studies, the decision-rule always produced a sparse and positive definite MAP-estimate for Θ, negating the need for an ad hoc correction to the eigenvalue structure of the estimate. This included tens of thousands of sparse MAP-estimates.

### Empirical Bayes method for sparse precision matrix estimation

There may be a special case when there is a reason to assume that just a few off-diagonal elements of the precision matrix are non-zero, similar to the matrix d) described at the beginning of the section 3. The previous subsection indicated that the Ledoit and Wolf estimator is able to produce low risk estimates for Θ in general.

The Ledoit and Wolf estimator is a ridge-type estimator of the form *ρ*_1_ *I* + *ρ*_2_*S* where *ρ*_1_ and *ρ*_2_ are specific parameters determined by the data (for more details, see [19]). It can be seen that all the ridge-type estimators of the above-mentioned form have a Bayesian interpretation of the form Σ ∼ *W*^−1^(*n*/*ρ*_2_ − *n* + *p* + 1, *nρ*_1_/*ρ*_2_ *I*) for the covariance matrix which will lead to the ridge-type estimate for the posterior mean—or for the MAP in the similar matter. The prior for the precision matrix is the Wishart prior with *ρ*_2_/*nρ*_1_ *I* set as the scale matrix. Thus, we use the Ledoit and Wolf estimator as an empirical Bayes estimator for Θ. Our goal is to improve the estimator of the precision matrix in the sparse setting by using our decision-rule procedure with the Ledoit and Wolf estimator. In this chapter we examine the performance of this approach when *p* > *n* and refer to the Ledoit and Wolf estimator with our decision-rule procedure shortly as an empirical Bayes estimate.

To examine this setting, sparse matrices with dimensions 20, 50 and 100 were created with at least 80% off-diagonal elements set to zero, as described at the beginning of the section. 50 different data sets were simulated. Again, both empirical Bayes and graphical lasso estimates were calculated to compare methods. Because of the poor performance of the EBIC with Glasso in the risk minimization setting in the previous chapter, we used the 5-fold cross-validation described in [8] to choose the regularization parameter for Glasso. The classification diagnostics are presented in Table 1. We also tested the EBIC as the penalization parameter selection criterion for Glasso but this resulted in high frequentist risk estimates (results not shown) compared to the ones presented in Table 1.

**Table 1:** Simulation results for different precision matrices.

p	n	Glasso			Empirical Bayes		
		K-L	L2	Ql	K-L	L2	Ql
**20**	**10**	2.88 (0.48)	2.33 (0.15)	12.14 (10.93)	2.20 (0.97)	1.98 (0.38)	38.39 (51.69)
	**13**	2.91 (0.86)	2.34 (0.40)	12.39 (28.05)	1.76 (0.52)	1.84 (0.19)	16.17 (23.10)
	**15**	2.52 (0.39)	2.23 (0.17)	9.10 (8.16)	1.63 (0.35)	1.79 (0.11)	11.71 (13.56)
	**19**	2.63 (0.81)	2.26 (0.37)	8.73 (15.24)	1.54 (0.20)	1.76 (0.06)	8.45 (8.53)
**50**	**20**	8.98 (0.51)	5.00 (0.18)	37.77 (33.37)	8.20 (0.59)	4.58 (0.11)	54.64 (41.60)
	**30**	7.56 (0.48)	4.68 (0.16)	28.15 (24.92)	7.80 (0.61)	4.59 (0.17)	31.82 (30.30)
	**35**	6.96 (0.42)	4.56 (0.16)	26.49 (19.57)	7.73 (0.74)	4.59 (0.17)	30.83 (28.67)
	**40**	6.64 (0.32)	4.51 (0.16)	29.25 (21.81)	7.72 (0.69)	4.62 (0.16)	24.46 (21.54)
**100**	**50**	20.67 (0.70)	9.02 (0.17)	66.00 (45.98)	24.53 (3.15)	9.29 (0.26)	81.56 (117.82)
	**60**	19.05 (0.59)	8.79 (0.18)	65.97 (45.69)	22.56 (1.91)	9.03 (0.21)	50.80 (62.98)
	**70**	17.45 (0.52)	8.51 (0.16)	54.55 (29.61)	21.14 (0.76)	8.89 (0.13)	34.54 (40.68)
	**90**	15.07 (0.38)	8.07 (0.12)	54.72 (27.42)	19.45 (0.61)	8.57 (0.13)	25.70 (29.73)

Risk measures based on the frequentist means and standard deviations of Kullback-Leibler (K-L), L2 and quadratic (Ql) losses for graphical lasso (Glasso) and empirical Bayes (Ledoit and Wolf) estimator combined with our decision-rule.

The performance of the Glasso improves substantially when using the 5-fold cross-validation. Now the Glasso is able to give comparable results and even outperform our empirical Bayes estimate. The Glasso seems to have some problems with consistency in this setting because the risk estimates appear not to decrease as the sample size increases. Still our empirical Bayes method gives lower risk in some of the cases and seems to be consistent as the sample size increases. Again, the decision-rule always produced a positive definite estimate with no need to tamper with the final sparse estimate. Because the Ledoit and Wolf estimator does not need any iteration whatsoever, the empirical Bayes estimate can be calculated almost instantly and the stepwise decision takes about 20 seconds to be executed when *p* = 100.

**Fig 7:**
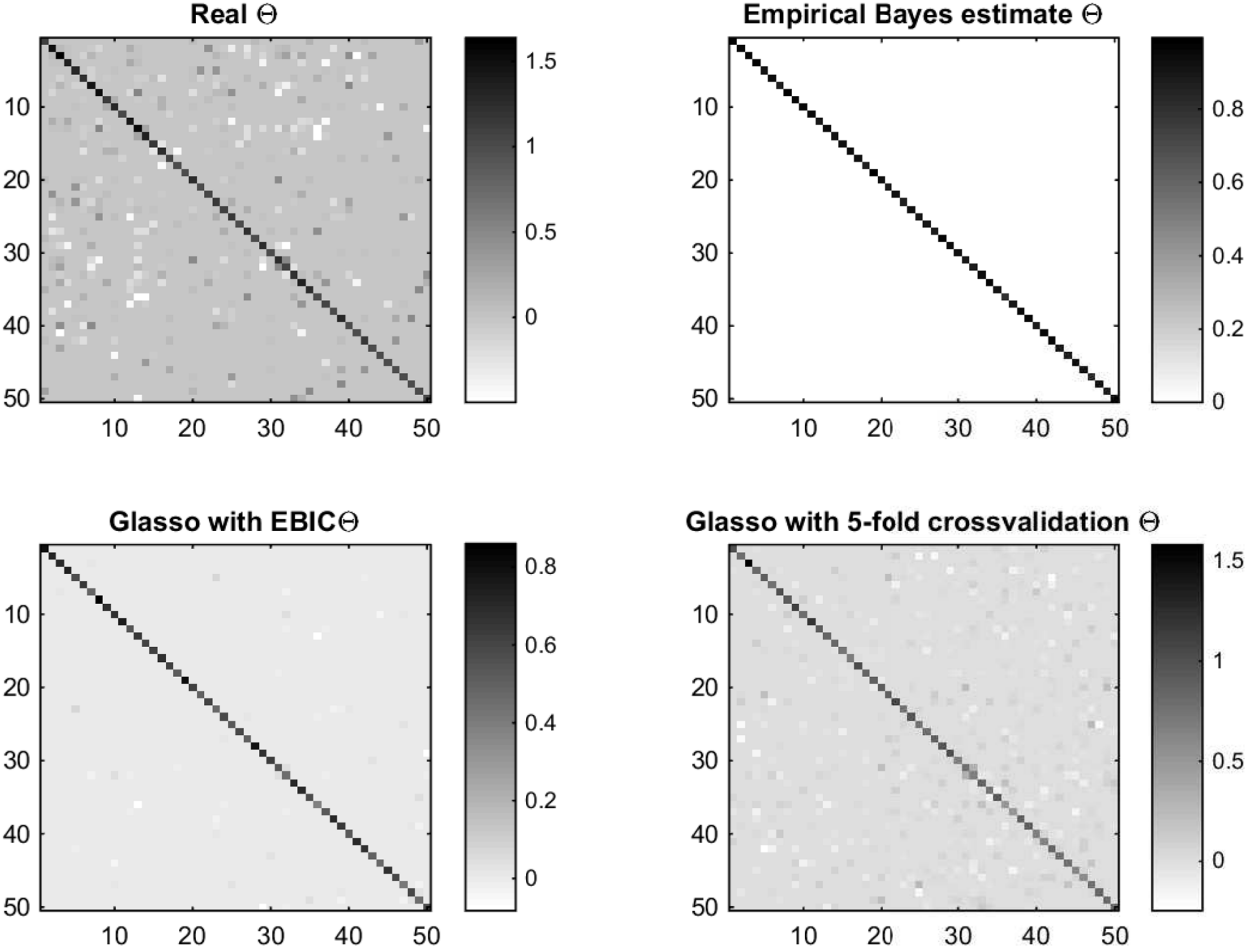
Image plots of the precision matrices. From left to right at the first row: The image plot of the real Θ and empirical Bayes estimate. On the second row: Graphical lasso with the penalization parameter *ρ* chosen with EBIC and graphical lasso with *ρ* chosen with 5-fold cross-validation when *p* = 50 and *n* = 40.

The choice between EBIC and cross-validation is a trade off between two features. Glasso with EBIC produces way more sparse structure for the estimate of Θ than with the cross-validation. As mentioned by [21], the cross-validation tends to produce dense graphs with many false positive edges. This is not desirable if the Glasso is used in the graph estimation in high dimensions. As illustrated in Fig 7, the Glasso with 5-fold cross-validation seems to be closer to the real structure of the Θ and the Glasso estimate with EBIC has just few non-zero off-diagonal elements. On the other hand, the empirical Bayes estimate produces way too sparse estimate with no sign of the structure of the true precision matrix with this data set. In the next section we will see that this “over-sparsity” is not a problem when *n* ≫ *p*.

## Application to real data

In this chapter, we extend previously examined estimates for the precision matrix to the graph estimation in a real data example. First we introduce some concepts needed for the graph construction.

Denote that G=(N,E) is a graphical model with node set N={1,…,p} and edge set E in N×N. One can parallel the nodes with the variables of interest. We estimate the graph associated with the GGM by using the elements of the precision matrix to compose a symmetric adjacency matrix. If $\hat{\Theta }$ is an estimate of the precision matrix, one can construct an adjacency matrix *Ad*, (*Ad*_*ij*_) ∈ {0, 1}, *i*, *j* = 1, …, *p*, in the following manner.

$$\begin{array}{c} Ad_{ij}=\left\{\begin{array}{cc} 1, & \text{if}\;\Theta_{ij}\neq 0.\\ 0, & \text{otherwise}. \end{array}\right. \end{array}$$ (5)

The pair (*i*, *j*) is contained in the edge set E if and only if the element *Ad*_*ij*_ is non-zero. The diagonal of the adjacency matrix can be set as zero so that there are no pairs (*i*, *i*) in the edge set. To illustrate the estimated graph $\hat{G}$, we drew a edge between the nodes (variables) (*i*, *j*), *i* ≠ *j* if the element *Ad*_*ij*_ is one. Otherwise there is no edge between the nodes and we posit that the variable *i* is conditionally independent of the variable *j* given all remaining variables (see for example [26]). For this reason, one only needs to examine either the upper or the lower diagonal elements of the adjacency matrix *Ad*.

For reasons of clarity, a flow cytometry dataset from [13] is analyzed. A data frame from R-package “gss” (https://cran.r-project.org/web/packages/gss/index.html) containing 7466 cells, with flow cytometry measurements of 11 phosphorylated proteins and phospholipids on the log_10_ scale of the original, is used. The networks were inferred with our decision-rule applied to various aforementioned Bayes estimates, Glasso and Meinshausen and Bühlmann approximation (MB approximation) [27] contained in the “glasso” package. The results were compared to the undirected graph presented in [4]. A directed acyclic graph of the data can be found in [3], and an undirected graph similar to ours from [4]. Unlike [4], we used standardized data and did not precorrect the systematic effects from the data. The precorrection could reduce some edges between the nodes in the network with graphical lasso.

We applied our decision-rule to three different Bayesian Wishart based methods; our MAP-estimate with diminished condition number, Ledoit and Wolf estimate and the posterior estimate defined according to the model 1 of Bouriga and Féron [20]. For the Metropolis-within-Gibbs estimation of Bouriga and Féron, we used 20000 MCMC iterations with 5000 as the burn-in period to determine the final posterior estimate using the MATLAB. Other analyses were done with R.

**Fig 8:**
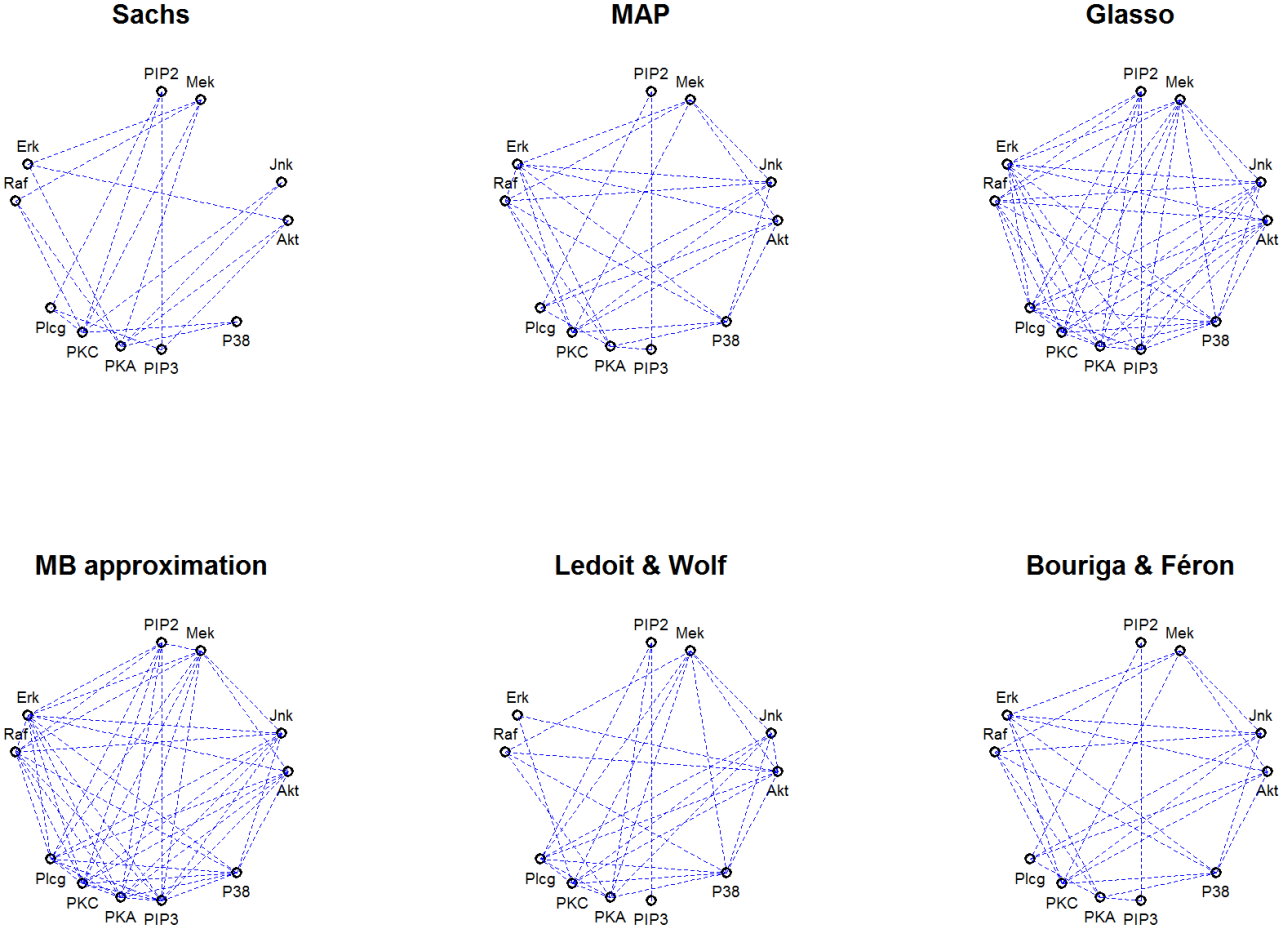
Undirected graphs from cell-signaling data. The original undirected graph from [13] (Sachs) and GGMs estimated with MAP with diminished condition number and decision-rule (MAP), graphical lasso (Glasso), Meinshausen and Bühlmann approximation (MB approximation), Ledoit and Wolf estimate with our decision-rule (Ledoit & Wolf) and posterior for Θ estimated with the model 1 introduced by Bouriga and Féron with our decision-rule (Bouriga & Féron).

For Glasso, we used the EBIC to choose the penalization parameter *ρ* from the sequence [0.01, 0.02, 0.03, …0.8] in the hope of attaining sparse graph. We used this same value in the MB approximation. The estimated graphs based on the precision matrix are shown in Fig 8.

We also investigated how the methods performed in the estimation of the graphical structure compared to the network of Sachs. For this, we computed the specificity, sensitivity, fall-out, precision and Matthew Correlation Coefficients (MCC) which are defined as follows:

Specificity=TN/(TN+FP) (6)

Sensitivity=TP/(TP+FN) (7)

Fall−out=FP/(FP+TN) (8)

Precision=TP/(TP+FP) (9)

$$MCC=\frac{\left(TP\times TN-FP\times FN\right)}{\sqrt{\left(TP+FP\right)\times \left(TP+FN\right)\times \left(TN+FP\right)\times \left(TN+FN\right)}},$$ (10)

in which *TN* is the number of true negatives, *FP* is the number of false positives, *TP* is the number of true positives and *FN* is the number of false negatives. The *MCC* can vary between −1 and 1. The closer the values of specificity, sensitivity, precision and MCC are to one, the better the classification is. For the fall-out (aka the false positive rate), which is the same as 1 − *specificity*, the value closer to zero is better. The results are presented in Table 2.

**Table 2:** Diagnostics for competing methods.

	MAP	Ledoit & Wolf	Bouriga & Féron	Glasso	M & B approximation
**Specificity**	0.583	0.528	0.611	0.222	0.250
**Sensitivity**	0.632	0.632	0.632	1.000	0.947
**Fall-out**	0.417	0.472	0.389	0.778	0.750
**Precision**	0.444	0.414	0.462	0.404	0.400
**MCC**	0.204	0.152	0.231	0.300	0.243

Summary of specificity, sensitivity, fall-out, precision and Matthew Correlation Coefficients (MCC) for the MAP-estimate with regularized condition number (MAP), Ledoit and Wolf estimate, Bouriga and Féron posterior estimate, graphical lasso (Glasso) and Meinshausen and Bühlmann approximation when the estimated graphs are compared to the “Sachs” graph.

Comparing graphs in the Fig 8 it appears that all of the Bayesian methods produce more sparse graphs than the Glasso and MB approximation. The diagnostics in the Table 2 indicate that the Bayesian approaches performed at least comparable manner to the frequentist competitors. We note, that the MCC is higher with the frequentist methods. On the other hand, these methods produce the highest fall-out and sensitivity, which is due to the dense graph estimates. From the practical point of view, the Bayesian methods produce graphs which are visually easier to examine. The MAP-estimate and the Bouriga and Féron estimate differ from each other by just one edge (the edge between variables ‘Erk’ and ‘Raf’). Overall the MAP-estimate with our decision-rule is able to detect false positive edges from the graph associated with this data set quite efficiently.

We noticed that with the default choice of the EBIC parameter *γ* = 0.5, the networks are quite dense. When we increased the *γ* over the value 0.6, it reduced the number of the estimated edges but beyond this point the increment of the parameter had no effect to the graph structure. The graphs presented in the Fig 8 are derived from the *γ* set as 0.6.

Glasso and MB approximation tend to favour dense graph structure and the adjustment of the parameter *γ* had whatsoever effect to the number of edges in the graph. This may be due to the weight of the likelihood which causes the Glasso to be close to the ML-estimate when *n* ≫ *p*. We also tried the analysis with the 5-fold cross-validation but the results were identical to those with regularization parameter chosen by EBIC.

The inferred networks drawn from the estimated precision matrices may be dense because there are several edges between nodes which are redundant, such as the node between PKC and Erk. From the original network of [13] we can see that both PKA and Erk are downstream of PKC and, thus, PKC is conditionally independent of Erk given PKA and other variables; that is, (PKC ⊥ Erk | other variables). Based on [13], there are also some unmeasured variables which cause indirect connections.

## Discussion

We have proposed improvements to the classic Bayesian estimates when using Wishart prior for the precision matrix by just increasing the degrees of freedom parameter of the Wishart prior. By monitoring the condition number of the estimate, we can determine an estimate with lower risk, without loss of computational speed. Apart from graphical lasso, analytical precision estimates for the matrix elements are available and can be calculated without iterative methods or MCMC sampling. When *p*/*n* ≪ 1, Wishart prior, combined with a decision-rule step where few additional elements are set to zero, may be a good choice for sparse precision and covariance matrix estimation.

The simulations with several sparsity patterns of the precision matrix indicate that there is no happy compromise between sparse estimate for the precision matrix and low risk estimate when measured with the loss-functions we have used. In the regression-based lasso approach, we know that the cross-validation does produce a model with a reliable prediction ability but this generally does not lend itself to a very sparse model [28] without some pre-modification of the data [4].

From this, a contradiction arises in terms of classic Bayesian analysis. When there are more data points, it is natural that the data starts to dominate over the prior and the posterior estimate comes closer to the MLE. This is troublesome because it may cause overly dense graphs when the true precision or covariance matrix is sparse. However, as can be seen from our example with the flow cytometry data, when *n* ≫ *p*, our decision-rule is able to produce a sparse graph whereas graphical lasso is able to produce only moderately sparse graph. Artificial adjustment of the penalization parameter *ρ* of the graphical lasso will produce a sparser network (cf. [8], [3], [4], [9]) but we wanted to avoid this kind of unjustified analysis. Also methods such as stability approach to regularization selection (StARS) [21] could be used with the graphical lasso. We tested this with the R package “huge” [29]. With StARS, the Glasso derived network was very sparse (just eight edges) with the following diagnostics: Specificity 0.944, sensitivity 0.316 fall-out 0.056, precision 0.750 and MCC 0.351. Clearly the performance of the Glasso depends on the procedure used to choose the regularization parameter.

It is possible to simulate independent posterior samples and obtain credible regions for the whole precision matrix because of the known analytic posterior distribution. In [4], credible region based thresholding is used to choose which off-diagonal elements should be set at zero; if the credible region contained a value of zero, the corresponding off-diagonal element was set as zero. We also tried this kind of strategy but found it to be inferior to EBIC-strategy with respect to both time and performance. Furthermore, this approach has the problem of choosing the width of the (1 − *α*)100% credible region. In our simulation study the 95% credible region was not an efficient way to set putative off-diagonal values to zero. They also used arbitrary 30% credible intervals in [4], which we found to be unsatisfactory in our own experiments. Additionally, in our Wishart setting the credible region-based approach does not guarantee that the sparse posterior estimate of Θ would be positive definite and, thus, some ad hoc modification would still be needed in order to obtain a positive definite estimate.

With documented experiments, a variety of different estimation approaches (including our approach) support the fact that there still does not exist an all-purpose procedure for covariance and precision matrix estimation in all problem settings. The real structure of the precision matrix and the choice of loss function significantly affect the final result, as noted by [20]. What makes stepwise thresholding a viable alternative is that it can be very fast in special cases, has no convergence problems typical for MCMC sampling, can work at a similar level to graphical lasso—or even better when *p*/*n* ≪ 1—and appears to be consistent when using the EBIC.

## Supporting Information

S1 Code Collection Collection of R codes used in this article. (7Z)
